# Severe Acute Respiratory Infection (SARI) sentinel surveillance in the country of Georgia, 2015-2017

**DOI:** 10.1371/journal.pone.0201497

**Published:** 2018-07-30

**Authors:** Giorgi Chakhunashvili, Abram L. Wagner, Laura E. Power, Cara B. Janusz, Ann Machablishvili, Irakli Karseladze, Olgha Tarkhan-Mouravi, Khatuna Zakhashvili, Paata Imnadze, Gregory C. Gray, Benjamin Anderson, Matthew L. Boulton

**Affiliations:** 1 National Centers for Disease Control and Public Health, Tbilisi, Georgia; 2 Department of Epidemiology, School of Public Health, University of Michigan, Ann Arbor, Michigan, United States of America; 3 Department of Internal Medicine, Division of Infectious Disease, University of Michigan Medical School, Ann Arbor, Michigan, United States of America; 4 Division of Infectious Diseases, School of Medicine, Global Health Institute, Duke University, Durham, North Carolina, United States of America; 5 Global Health Research Center, Duke Kunshan University, Kunshan, China; 6 Program in Emerging Infectious Diseases, Duke-NUS Medical School, Singapore; University of Calgary, CANADA

## Abstract

**Background:**

Severe Acute Respiratory Infection (SARI) causes substantial mortality and morbidity worldwide. The country of Georgia conducts sentinel surveillance to monitor SARI activity and changes in its infectious etiology. This study characterizes the epidemiology of SARI in Georgia over the 2015/16 and 2016/17 influenza seasons, compares clinical presentations by etiology, and estimates influenza vaccine effectiveness using a test-negative design.

**Methods:**

SARI cases were selected through alternate day systematic sampling between September 2015 and March 2017 at five sentinel surveillance inpatient sites. Nasopharyngeal swabs were tested for respiratory viruses and *Mycoplasma pneumoniae* using a multiplex diagnostic system. We present SARI case frequencies by demographic characteristics, co-morbidities, and clinical presentation, and used logistic regression to estimate influenza A vaccine effectiveness.

**Results:**

1,624 patients with SARI were identified. More cases occurred in February (28.7%; 466/1624) than other months. Influenza was the dominant pathogen in December-February, respiratory syncytial virus in March-May, and rhinovirus in June-November. Serious clinical symptoms including breathing difficulties, ICU hospitalization, and artificial ventilation were common among influenza A and human metapneumovirus cases. For influenza A/H3, a protective association between vaccination and disease status was observed when cases with unknown vaccination status were combined with those who were unvaccinated (OR: 0.53, 95% CI: 0.30, 0.97).

**Conclusions:**

Multi-pathogen diagnostic testing through Georgia’s sentinel surveillance provides useful information on etiology, seasonality, and demographic associations. Influenza A and B were associated with more severe outcomes, although the majority of the population studied was unvaccinated. Findings from sentinel surveillance can assist in prevention planning.

## Introduction

Severe Acute Respiratory Infection (SARI) is an important cause of morbidity and mortality worldwide. Increased uptake of the pneumococcal conjugate vaccine (PCV) and the *Haemophilus influenzae* type b (Hib) vaccine have led to declines in fatal outcomes due to lower respiratory infections, from 3.4 million deaths worldwide in 1990 to 2.8 million in 2010.[1] However, SARI is associated with a large number of different viral and bacterial agents, notably influenza A and B viruses, parainfluenza viruses, coronaviruses, respiratory syncytial viruses (RSV), adenoviruses (AV), and rhinoviruses.[1]

The complex interrelationships between the prevalent pathogens that cause SARI are not well understood.[2] Influenza, in particular, is a very common and sometimes serious infectious disease, with an epidemiological picture marked by seasonal peaks in winter months and antigenic drift and shift, which potentiate local and regional outbreaks and global pandemics. A community influenza burden assessment in England found that approximately 18% of the unvaccinated population became infected with influenza A or B viruses each season during 2006–2011.[3] Although three-quarters of the infections in this population were asymptomatic,[3] symptoms and complications are often more severe among well recognized high-risk groups. For example, the hospitalization rate due to respiratory illness during an influenza season in Japan was 4.3 times higher among pregnant women than among those who were not pregnant.[4]

Routine monitoring of influenza A/B virus activity is the foundation for preparedness and response to the virus and its pandemic potential. Special studies are not sufficient to provide timely and actionable information to guide response. In 2006, WHO and other organizations evaluated the influenza A pandemic response and preparedness capacity in the country of Georgia, among other countries. The review found deficiencies in the local epidemiological and laboratory capacity.[5] In response, a surveillance system for SARI was established to provide improved epidemiologic monitoring of influenza and other respiratory disease within the country of Georgia.[6]

According to previously modeled estimates regarding burden of SARI among children 0–4 years in Georgia,[7] influenza A virus and RSV are associated with 32% and 54% of all acute lower respiratory illnesses, respectively. Epidemiologic characterization of influenza among high-risk groups in Georgia has not been previously reported in the literature. This study aimed to characterize the seasonality and epidemiology of SARI in the country of Georgia over two influenza seasons (2015–2016 and 2016–2017), to describe the etiological and clinical patterns observed, and to assess seasonal influenza vaccine effectiveness using a case-test negative design.

## Materials and methods

### Sentinel surveillance

Georgia’s sentinel-based SARI surveillance network collects epidemiologic, clinical and virologic data on patients who present with Severe Acute Respiratory Infection (SARI) at five clinical inpatient sites. One site is located in Tbilisi, the country’s capital which is in the eastern region of the country, and the four other sites are located in Kutaisi, the second largest city in the country’s central-west region.

Case selection followed the WHO Global Epidemiological Surveillance Standards for Influenza [8] with cases enrolled through an alternate day, systematic sample method. SARI cases were defined as a respiratory infection with fever of ≥ 38°C, cough, onset within the last 10 days, and hospitalization.[8]

Hospitalized patients with SARI provided demographic information, including age, sex, and residence through questionnaires. The surveillance system also collects patients’ self-reported history of seasonal influenza vaccination within the past year in response to the question, “Are you vaccinated against influenza?” Participants who responded affirmatively were subsequently questioned about when they were vaccinated, and were only recorded as having received the vaccine if it was received specifically for the on-going season. Based on date of sampling and enrollment into the study, cases of SARI captured in the sentinel surveillance system between September 29, 2015, and March 15, 2017, were included in this present manuscript.

### Specimen collection and molecular characterization

A single pharyngeal and nasal swab were collected from each patient at the time they were diagnosed as a SARI case by the physician. After collection, the nasal and pharyngeal swabs were inserted into the same 2 ml cryovial containing viral transport medium. The swabs were expressed on the side of the cryovials and broken off into the cryovials. These specimens were stored in an indexed cryovial box inside a cooler of wet ice for shipment to the laboratory at Richard Lugar Center for Public Health Research in Tbilisi, Country of Georgia, where they were preserved at -80°C until molecular studies were performed.

The QIAamp Viral RNA Mini Kit (Qiagen, Hilden, Germany) were used for viral RNA and DNA extraction. To guarantee RNA integrity, samples were lysed under highly denaturing conditions to inactivate RNases. Alcohol was added and lysates loaded onto the QIAamp spin column. Viral RNA and DNA bind specifically to the QIAamp silica membrane while contaminants pass through. Pure viral RNA/DNA were eluted in a 60 μl buffer. Wash buffers removed impurities and pure, ready-to-use RNA/DNA was then eluted in water or low-salt buffer. Total nucleic acid extracts were stored at -80°C until further processing by PCR.

FTD Respiratory Pathogens 21 (Fast Track Diagnostics) multiplex Real-Time PCR diagnostic strategy was used to detect influenza A, pandemic influenza A/H1N1 (A/H1N1pdm09 or A/H1pdm09), influenza B, rhinovirus, coronavirus NL63, 229E, OC43, HKU1, parainfluenza 1, 2, 3, 4, human metapneumovirus A/B, bocavirus, respiratory syncytial virus (RSV) A/B, adenovirus, enterovirus, parechovirus, and *Mycoplasma pneumoniae*. The CFX96™ Real-time PCR System (Bio-Rad, Hercules, CA, USA) that detects five different fluorescent dyes simultaneously was used for multi-pathogen detection in one clinical sample. To identify A/H3 virus, we used a real-time RT-PCR Influenza A subtyping kit donated by the US CDC.

### Descriptive and statistical analysis

Frequencies and proportions of SARI patients were calculated by demographic characteristics, comorbidities (some patients had more than one), and clinical presentation. To portray the seasonal distribution of the different etiologic pathogens, the weekly case count by pathogen is depicted graphically for each pathogen, and the proportion of SARI patients who were diagnosed with each pathogen was calculated for each month.

We used a case-test negative design to estimate the effectiveness of seasonal influenza vaccination within the past year.[9] An odds ratio (OR) was calculated comparing the odds of being positive for influenza among those with and without a prior seasonal influenza vaccination. This odds ratio was calculated separately for the A/H1pdm09 and A/H3 strains, along with any influenza virus strain. The reference controls (“test negatives”) were those SARI cases who were not positive for any influenza virus. Many patients had an unknown vaccination status. As a sensitivity analysis, the OR was calculated three separate ways: not including any patient with unknown vaccination status, and then including vaccination unknowns into either the vaccinated or unvaccinated group. The OR was estimated using a logistic regression model that also controlled for sex and age and was limited to individuals ≥1 year of age.

The dataset was analyzed in SPSS version 20 (IBM Corporation, Armonk, NY, USA).

### Ethics statement

Data were de-identified prior to analysis. Because the analysis was part of regular public health surveillance activities, it was deemed exempt from ethical oversight by the Georgia NCDC Institutional Review Board.

## Results

A total of 1,624 patients with SARI were identified by the sentinel surveillance system from September 29, 2015, to March 15, 2017 (Table 1). More SARI cases were <1 year (22%; 356/1624) or 1–4 years of age (28%; 449/1624) than in other age groups. Only 9% (150/1624) of cases were among persons ≥65 years. Slightly more cases were male (54%; 877/1624) than female, and 3% (52/1624) were pregnant women. Comorbidities were found in 19% (315/1624) of the SARI cases, the most common being cardiovascular disease (10%; 158/1624), diabetes (4%; 68/1624), respiratory disease (4%; 66/1624), and neurological disease (4%; 61/1624). Many SARI patients received clinical care such as hospitalization in an intensive care unit (ICU) (37%; 606/1624), artificial ventilation (12%; 189/1624), or antiviral treatment (21%; 338/1624). Few deaths (1%; 14/1624) were recorded among the SARI cases.

**Table 1:** Demographic and clinical characteristics of cases within a Severe Acute Respiratory Infection surveillance system in the country of Georgia, 2015–2017.

	All SARI cases	InfV A/H1	InfV A/H3	InfV B	Any InfV	PIV	CoV	BoV	RV	EV	AV	MPV	RSV	Any co-inf	Neg
**Overall**	1624	331	134	77	542	75	87	78	195	18	84	40	249	222	489
**Age**															
<1 year	356 (22%)	8%	12%	10%	9%	29%	31%	28%	31%	22%	26%	30%	51%	32%	17%
1–4 years	449 (28%)	24%	26%	23%	24%	37%	33%	54%	41%	50%	50%	18%	31%	43%	21%
5–14 years	232 (14%)	12%	15%	23%	14%	13%	14%	8%	17%	22%	8%	18%	10%	14%	17%
15–29 years	165 (10%)	12%	13%	29%	15%	3%	7%	1%	3%	6%	6%	20%	3%	3%	11%
30–64 years	262 (16%)	33%	18%	9%	26%	9%	9%	4%	2%	0%	6%	13%	3%	5%	19%
≥65 years	150 (9%)	10%	16%	5%	11%	4%	5%	5%	5%	0%	2%	3%	2%	3%	14%
**Sex**															
Male	877 (54%)	58%	47%	45%	54%	59%	60%	59%	54%	33%	61%	45%	56%	55%	53%
Female	743 (46%)	42%	53%	55%	46%	37%	40%	41%	45%	67%	38%	55%	44%	44%	47%
Pregnant	52 (3%)	5%	7%	6%	6%	1%	0%	0%	1%	0%	4%	5%	0%	1%	3%
Postnatal period	23 (1%)	1%	7%	3%	3%	3%	0%	0%	2%	6%	1%	0%	0%	1%	0%
**Comorbidity**															
None	1294 (80%)	74%	70%	90%	75%	83%	89%	86%	89%	100%	89%	85%	90%	89%	75%
Yes	315 (19%)	26%	29%	10%	25%	13%	9%	13%	11%	0%	8%	15%	10%	9%	24%
Respiratory	66 (4%)	4%	10%	4%	5%	4%	2%	4%	2%	0%	1%	3%	2%	2%	5%
Asthma	14 (1%)	2%	2%	0%	1%	1%	0%	0%	0%	0%	0%	0%	0%	0%	1%
Diabetes	68 (4%)	6%	10%	1%	7%	3%	1%	1%	1%	0%	4%	3%	1%	1%	4%
Cardiovascular	158 (10%)	12%	16%	4%	12%	11%	6%	6%	7%	0%	2%	8%	5%	5%	12%
Obesity	26 (2%)	3%	3%	1%	3%	0%	0%	0%	1%	0%	0%	3%	0%	0%	2%
Kidney disease	21 (1%)	1%	5%	0%	2%	0%	2%	1%	1%	0%	0%	0%	0%	0%	2%
Liver disease	31 (2%)	3%	1%	4%	3%	1%	1%	1%	0%	0%	0%	3%	1%	1%	3%
Neurological	61 (4%)	4%	4%	4%	4%	4%	5%	3%	3%	0%	4%	8%	4%	4%	4%
Immunodeficiency	4 (0%)	0%	0%	0%	0%	1%	1%	0%	0%	0%	0%	0%	0%	0%	0%
**Disease presentation**															
Fever	1539 (95%)	95%	98%	95%	96%	93%	97%	96%	95%	100%	94%	100%	96%	95%	92%
Cough	1392 (86%)	86%	94%	90%	88%	81%	89%	95%	88%	100%	89%	95%	93%	90%	77%
Breathing difficulty	1213 (75%)	76%	80%	69%	76%	64%	68%	78%	80%	72%	80%	93%	74%	73%	72%
**Clinical outcomes**															
Hospitalized ≥1 night	1545 (95%)	96%	93%	96%	95%	95%	94%	99%	96%	89%	96%	98%	98%	96%	93%
Hospitalized in ICU	606 (37%)	44%	40%	21%	39%	33%	30%	21%	39%	28%	26%	50%	32%	31%	40%
Artificial ventilation	189 (12%)	18%	13%	0%	14%	12%	5%	4%	9%	0%	7%	15%	4%	6%	14%
Antiviral treatment	338 (21%)	40%	28%	12%	33%	7%	6%	12%	7%	6%	14%	25%	6%	9%	24%
Died	14 (1%)	1%	3%	0%	1%	0%	1%	0%	1%	0%	2%	0%	0%	0%	1%
**Seasonal influenza vaccination**															
Yes	20 (1%)	1%	6%	0%	2%	1%	1%	0%	1%	6%	0%	0%	0%	1%	1%
No	1376 (85%)	75%	89%	97%	81%	83%	87%	97%	91%	83%	94%	98%	95%	92%	80%
Unknown	203 (12%)	24%	4%	0%	15%	12%	9%	1%	7%	11%	4%	3%	4%	5%	17%

AV, adenovirus; BoV, bocavirus; co-inf, co-infection; CoV, coronavirus; EV, enterovirus; ICU, intensive care unit InfV, influenza virus; Neg, negative for influenza and multiplex tests; MPV, metapneumovirus; PIV, parainfluenza virus; RSV, respiratory syncytial virus; RV, rhinovirus

Overall, 14% (222/1624) of individuals were co-infected with at least two pathogens. The most common pathogens characterized by co-infection were: RSV (39%, 87/222), rhinovirus (38%; 84/222), and influenza (20%; 44/222 [29 A/H1pdm09, 9 A/H3, and 6 B]). Compared to the entire population of SARI cases, co-infected individuals tended to be younger with 32% (71/222) aged <1 year and 43% (95/222) aged 1–4 years, compared to 22% (356/1624) and 28% (449/1624), respectively, for all SARI cases. Among the co-infected children ≤4 years 31% (69/222) were hospitalized in the ICU, compared to 37% (606/1624) hospitalized overall, and 6% (13/222) were supported with artificial ventilation, compared to 12% (189/222) overall.

### Seasonality

The number of reported SARI cases peaked in the month of February (29%; 466/1624), of which 49% (227/466) tested positive for influenza (Table 2). Although influenza A predominated earlier in the year, by March, influenza B was more common (present in 9% [15/171] of patients, versus 6% [11/171] of those with A/H1pdm09 and 2% [3/171] of those with A/H3). The most commonly identified pathogen in SARI patients was RSV in March (31%, 37/86) through May (24%, 6/25) and rhinovirus in June (28%, 13/46) through November (30%, 26/88). By December, influenza A/H3 was the most commonly identified pathogen, present in 30% (75/250) of patients. Patients with no identifiable pathogen, e.g. who were not positive for influenza or any of the pathogens in the multiplex test, were relatively common in the summer and early fall, comprising the majority of patients in August (69%, 11/16) and September (52%, 15/29).

**Table 2:** Monthly distribution of respiratory pathogens in a Severe Acute Respiratory Infection surveillance system in the country of Georgia, 2015–2017.

	Jan	Feb	Mar	Apr	May	Jun	Jul	Aug	Sep	Oct	Nov	Dec
Overall count	372	466	171	86	25	46	20	16	29	55	88	250
Influenza A/H1p	36%	37%	6%	0%	0%	0%	0%	0%	0%	0%	0%	6%
Influenza A/H3	9%	2%	2%	7%	4%	0%	0%	0%	0%	0%	3%	30%
Influenza B	1%	9%	9%	8%	12%	4%	0%	0%	0%	0%	0%	0%
Any influenza	47%	49%	17%	15%	16%	4%	0%	0%	0%	0%	3%	36%
Parainfluenza	4%	2%	1%	0%	4%	0%	0%	0%	7%	18%	14%	10%
Coronavirus	2%	3%	8%	10%	0%	2%	15%	13%	3%	24%	10%	7%
Human bocavirus	3%	3%	3%	3%	4%	4%	15%	0%	0%	5%	11%	10%
Rhinovirus	6%	5%	12%	15%	16%	28%	20%	19%	41%	38%	30%	13%
Enterovirus	0%	0%	1%	1%	0%	11%	5%	13%	14%	0%	1%	0%
Adenovirus	5%	3%	4%	2%	16%	4%	15%	0%	0%	7%	14%	6%
Human metapneumovirus	2%	2%	1%	13%	4%	9%	5%	0%	0%	4%	1%	1%
Respiratory syncytial virus	13%	15%	31%	43%	24%	4%	10%	0%	0%	2%	8%	10%
Any co-infection	9%	7%	19%	24%	24%	11%	35%	13%	21%	20%	24%	18%
Negative all tests	28%	26%	43%	21%	36%	43%	35%	69%	52%	24%	36%	26%

**Fig 1:**
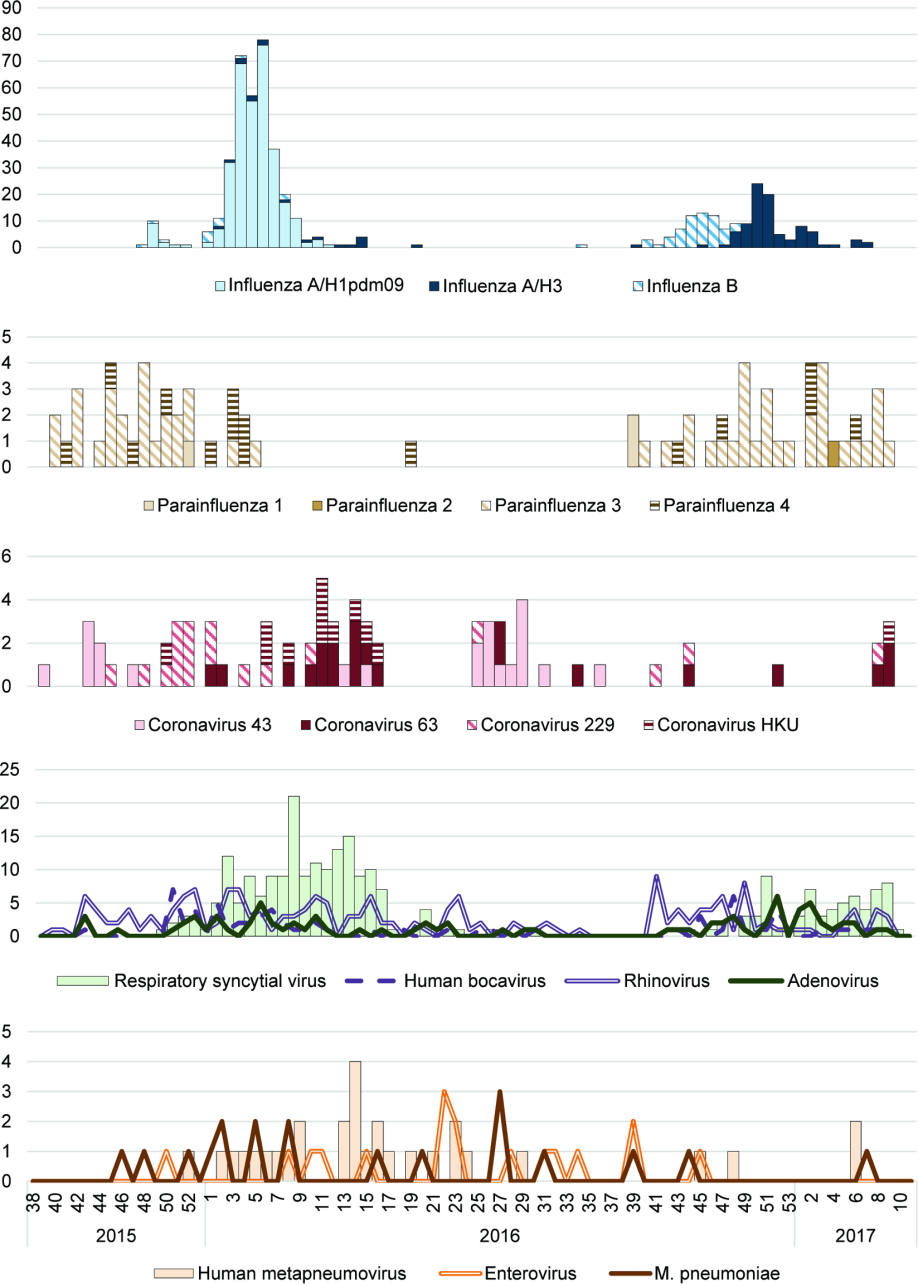
Weekly number of cases by pathogen from an inpatient Severe Acute Respiratory Illness surveillance system in the country of Georgia.

Overall, influenza was common in the initial weeks of the year, with A/H1pdm09 dominating in the 2015–2016 season, and A/H3 dominating in the 2016–2017 season (Fig 1). The number of influenza cases was also much higher in the 2015–2016 season than the 2016–2017 season (Fig 1). Parainfluenza was more common before the rapid increase in influenza cases, and coronavirus showed a less distinct seasonal pattern, with some cases apparent throughout the year, even in the summer. RSV was more common later in the spring when the number of influenza cases declined.

### Clinical presentation

Fever, cough, and breathing difficulty were the most common symptoms in SARI cases; fever and cough are included in the SARI case definition (Table 1). Overall, 75% (1,213/1,624) of SARI cases had breathing difficulty, especially in those with human metapneumovirus (93%, 37/40), adenovirus (80%, 67/82), rhinovirus (80%, 156/193), and influenza A/H3 (80%, 107/133) infections. Clinical outcomes associated with disease severity, including hospitalization in an ICU and artificial ventilation, followed similar patterns: hospitalization in an ICU was highest among those with human metapneumovirus (50%, 20/40) and influenza A/H1pdm09 (44%, 144/327); it was relatively low among those with adenovirus (26%, 22/82). Artificial ventilation was more common in those with influenza A/H1pdm09 (18%, 58/328), human metapneumovirus (15%, 6/40), influenza A/H3 (13%, 18/133), and parainfluenzavirus (12%, 9/72), and less than 10% for those with other infections.

Among the 14 individuals who died, 4 had influenza A/H3, 2 had influenza A/H1pdm09, 2 had rhinovirus, 2 had adenovirus, 1 had coronavirus 63, and 3 were negative for all pathogens tested for. No co-infections were identified among these individuals.

### Influenza vaccination

**Fig 2:**
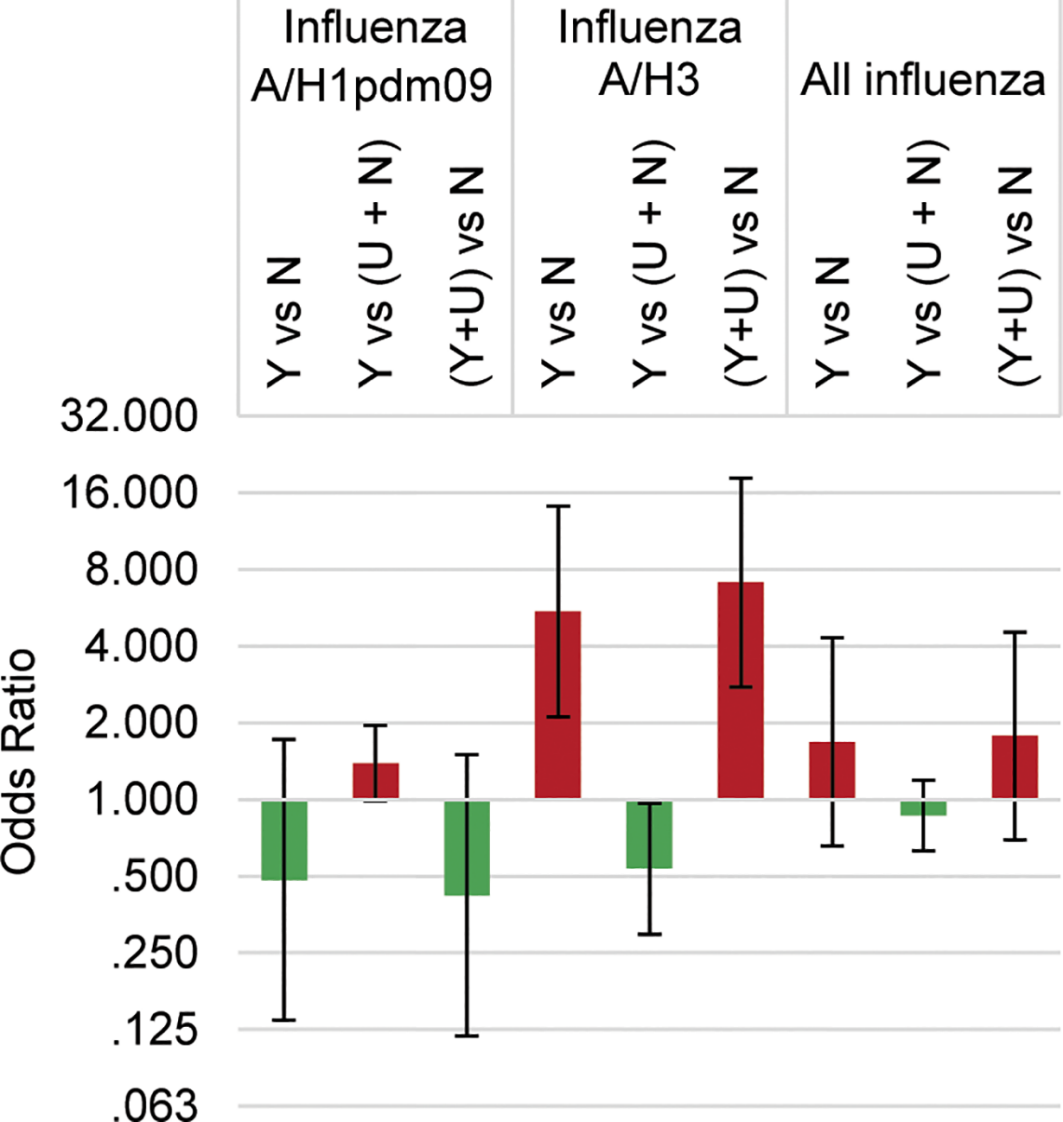
The odds of having influenza after receiving a seasonal influenza vaccination, according to a case-test negative design. Y: known to have received vaccine, U: unknown whether received vaccine, N: known to have not received vaccine.

A very small proportion of SARI cases reported influenza vaccination (1%; 20/1624) for the given season and 12% (206/1624) of cases reported unknown vaccination status. No individual with influenza B infection reported prior vaccination. The odds of being an influenza case compared to being a SARI case that did not have influenza varied by influenza strain and by how those with unknown vaccination status were categorized (Fig 2). For influenza A/H3, vaccination was associated with higher odds of influenza when excluding SARI cases with unknown vaccination status (OR: 5.47, 95% CI: 2.11, 14.18) or by combining those who were vaccinated with the unknown vaccination status group (OR: 7.11, 95% CI: 2.76, 18.29). The opposite trend, a protective association between vaccination and disease status was observed when cases with unknown vaccination status were combined with those who were known to be unvaccinated (OR: 0.53, 95% CI: 0.30, 0.97).

## Discussion

Sentinel surveillance has generated timely information on the etiologic and clinical patterns associated with SARI over the past two years in the country of Georgia. Routine monitoring of infectious etiologies associated with SARI at the local-level is critical for signaling epidemiologic and etiologic trends that may differ from one country to another. For example, a study of SARI patients in a number of countries of eastern Europe and western and central Asia found that the proportion of cases positive for influenza varied widely between country and season, from 2.1% in Armenia in 2011–2012 to 100% in Albania in 2009–2010.[10] And in the United States, surveillance of adults[11] and children[12] have been used to calculate the incidence of community-acquired pneumonia and the relative burden of different pathogens. The early detection of unusual or divergent activity from one country to another via the sentinel surveillance system can better prepare a country for emergency response needs and inform routine programmatic actions.

During 2015–2017 in Georgia, the predominant causes of SARI varied seasonally and depending on the month were influenza A/B, RSV and rhinovirus, although other viruses–coronavirus, parainfluenzavirus, adenovirus–were also present. The most serious clinical symptoms were found in those infected with influenza A and human metapneumovirus. A cross-country risk assessment of SARI activity among European countries during the 2015–2016 found a predominance of influenza A circulation in the region, with an uptick in severe disease in the earliest parts of the season,[13] which aligns with the patterns captured by the surveillance system in Georgia. While the distribution of infectious etiologies could change within and between seasons due to increased uptake of PCV, Hib and seasonal influenza vaccination, there was no evidence that circulating influenza viruses changed antigenically compared to the influenza vaccine virus for the same season.[13]

Another important aim of strengthening the SARI sentinel surveillance in the country of Georgia is to track the occurrence of SARI among high-risk groups. From 2015–2017, one-fifth of SARI cases had underlying comorbidities with cardiovascular disease observed in 10% of all SARI cases. The proportion of cardiovascular related comorbidities among influenza positive SARI cases was higher than all-cause SARI cases. Another special study on influenza mortality in Georgia conducted between 2009–2011 found that diagnosed coronary heart disease was commonly associated with fatal outcomes among influenza positive SARI cases (19% for type A, 68% for type B).[14] Individuals with cardiovascular disease could be targets for influenza vaccination programs in the future.

The seasonality of viral respiratory SARI observed in the 2015–2017 period in Georgia was similar to patterns observed in other temperate climate settings, where influenza circulation peaks in winter;[15] however, in contrast to this study where RSV was more commonly identified in the fall, we found RSV most common in the spring.[15] Observed trends in seasonality during the 2015–2017 season in Georgia are consistent with previously reported seasonality and age-distribution of disease among children.[13] Although we did not present detailed clinical characteristics of SARI which may be associated with specific pathogens, our findings provide evidence for the association between infection with specific respiratory viruses and severe clinical outcomes. Such information is helpful to clinicians in making more rapid diagnoses for the administration of treatment and patient management.

Only a few of the SARI cases reported during the 2015–2017 period had received the influenza vaccine. A study of pediatric influenza cases from the 2010–11 influenza season in Georgia also found low vaccination coverage–there being no case with a prior vaccination.[16] The lack of strong physician recommendations for influenza vaccination to the general population or even high risk groups are likely responsible for poor vaccine uptake. For example, a recent study found that only 43% of obstetrician-gynecologists in Georgia have recommended influenza vaccine to pregnant women, and only 18% reported vaccinating pregnant patients in the last influenza season.[17] Misinformation or knowledge gaps may also be at the root of hesitant recommendations from the healthcare worker community. For instance, one study of hospital-based physicians and nurses found that few were able to correctly identify influenza as the virus associated with H5N1 outbreaks in neighboring countries.[18] Analysis of risk factors associated with severe outcomes and continued monitoring of the etiologic nature of SARI occurrence in Georgia should provide an important evidence basis to help healthcare workers target high-risk groups and bolster seasonal influenza vaccine uptake in the population.

This study was not powered to detect an association between influenza vaccination status and laboratory-confirmed influenza. However, the test-negative study design presented in this analysis could provide a useful framework for annual evaluation of the effectiveness of the seasonal influenza vaccination efforts in the country of Georgia. Importantly, revisions to the existing data collection instruments for defining vaccination status to clearly identify those who received the current season vaccine could better assess the vaccine’s effectiveness from season to season.

### Strengths and limitations

Although we had a case definition of SARI established by the WHO, physicians reporting cases into the surveillance system did include a small number of cases who did not meet all symptom criteria. It is not clear how representative these findings are of SARI for the whole of Georgia. A longitudinal study in England estimated that only 5% of symptomatic influenza cases are medically attended and reported into the surveillance system.[3] Nonetheless, we are able to provide information over two influenza seasons (2015–2016 and 2016–2017) from a sentinel surveillance system which captures individuals throughout the country of Georgia. Additionally, we attempted to model the effectiveness of the influenza vaccine through a case-test negative design, however, we did not have the statistical power to test this by year. Overall, this study employed a rigorous sampling methodology and was able to enroll individuals throughout Georgia to describe the characteristics of SARI patients within the country.

## Conclusions

Continued strengthening of sentinel surveillance networks at the country and regional-levels is important to help shape policy and programmatic response to SARI activity while also keeping the public and media informed. Multi-pathogen diagnostic testing as part of Georgia’s SARI sentinel surveillance allows for in-depth understanding of SARI by specific etiology, seasonality of pathogens and case distribution by age. Influenza was a predominant infection associated with more severe outcomes, but the majority of the population studied was unvaccinated. The findings from this sentinel surveillance system can assist in improving preparedness for severe respiratory illness epidemics throughout the year and guide influenza vaccination policies for Georgia.
